# ATRA attenuate proteinuria via downregulation of TRPC6 in glomerulosclerosis rats induced by adriamycin

**DOI:** 10.1080/0886022X.2018.1456459

**Published:** 2018-04-05

**Authors:** Lei Zhang, Xiu-Ping Chen, He Qin, Ling Jiang, Yuan-Han Qin

**Affiliations:** a Department of Pediatric Nephrology, The First Affiliated Hospital of GuangXi Medical University, Nanning, China;; b Department of Pediatric, Affiliated Hospital of Hebei University, Baoding, China

**Keywords:** ATRA, glomerulosclerosis, proteinuria, TRPC6

## Abstract

**Objective:** In this research, we explored the molecular mechanism of proteinuria in glomerulosclerosis rats and the protective effects of ATRA.

**Methods:** This research set up three groups: SHO group, GS group, and ATRA group (15 mg/(kg d), Sigma, St. Louis, MO). The serum creatinine (Scr), urea nitrogen (BUN), and 24-h proteinuria were detected 12 weeks after administration of ATRA. The pathological and ultrastructure changes were observed under light microscope and transmission electron microscope. The protein expression of TGF-β_1_ and Col-IV in glomerulus was detected by immunohitochemistry method. The mRNA and the protein expression of glomerular TRPC6 were detected by RT-PCR and Western blot.

**Results:** In the rat model of GS, the expressions of TRPC6 were significantly elevated compared with the normal rat group; however, the use of ATRA down-regulated the expression of TRPC6 in the glomeruli and attenuated glomerulosclerosis and proteinuria. Scr and BUN were also improved by the treatment of ATRA.

**Conclusions:** Our results demonstrated that ATRA could ameliorate glomerulosclerosis and proteinuria in GS, which may be related to suppressed expression of TRPC6.

## Introduction

Proteinuria is a common clinical manifestation of kidney dysfunction, and is also a risk factor for the progression of chronic kidney disease [1]. Glomerular filtration barrier, including endothelial cells, basement membrane, and podocytes, play an important role in the development of proteinuria [2]. In recent years, the role of podocytes in glomerular proteinuria has received more and more attention. Numerous studies show that podocyte injury is a key factor in the development of proteinuria and podocyte slit diaphragm (SD) plays a pivotal role in maintaining the integrity of the glomerular filtration barrier [3,4]. TRPC6 is a recently discovered nonselective cation channel, mainly positioned at SD in podocyte [5]. It has been confirmed that TRPC6 interacts with SD structural proteins nephrin and podocin, implying an interaction between the podocyte structural molecules and the ion channels, and regulating structural molecules may help explicate the molecular mechanism of glomerular proteinuria pathogenesis [6]. All-trans retinoic acid (ATRA) is a pleiotropic drug that can alleviate a variety of renal disease, including minimal change disease, membranous nephropathy, focal segmental glomerulosclerosis (FSGS), and lupus nephritis. Our preliminary results also suggest that ATRA also has a preventive and therapeutic effect on glomerulosclerosis rats induced by adriamycin [7]. However, the mechanisms underlying the antiproteinuria effects of ATRA have not been well illuminated.

In this study, we measured TRPC6 expression levels in the adriamycin-induced glomerulosclerosis rats with or without ATRA treatments. Our purpose is to survey the effect of ATRA on dynamic changes of TRPC6 expressions in glomerulosclerosis rat model, in-depth understanding the molecular mechanism of glomerulosclerosis, and the special therapeutic effects of ATRA on proteinuria.

## Materials and methods

### Animal model and glomerular separation

All the programs were approved by the animal ethics committee of Guangxi Medical University. Male 8-week-old Wistar rats weighing 180 ± 20 g were provided by the Experimental Animal Center of Guangxi Medical University. Sixty rats were randomly divided into sham operation group (SHO), model group (GS), and ATRA group (ATRA), 20 rats in each group. One day before operation, ATRA group was given ATRA (Sigma, St. Louis, MO) 15 mg/(kg d) by gavage; SHO and GS groups were given isovolumic normal saline to the end of the experiment. Rats in the GS group and the ATRA group underwent uninephrectomy under the condition of sterile and a single tail vein injection of adriamycin (ADR) (WanLe Pharmaceutical Co., Ltd., Shenzhen, China) at a dose of 5 mg/kg 7 d after nephrectomy. Rats in the SHO group only underwent surgical exploration and a single tail vein injection of isovolumic saline on the seventh day. All animals survived after surgery.

At the end of 12 weeks, all rats were sacrificed by cervical dislocation, the kidney was removed, after renal capsule was removed, the renal cortex was separated, and 1% of the renal tissue was fixed with 10% neutral formalin. Taken one-third of the renal tissue cortex, cut into the size of about 1 mm^3^, moved to 150 mesh steel screen, gently rolling on ice, PBS washed repeatedly, then filtered suspension on overlapping 80 mesh and 220 mesh sieves, wash the 220 mesh sieve with PBS, collect the rinse solution, 800 r/min, centrifuge 3 min, collecting sediment. The remaining kidney tissue was frozen and stored at −80 °C.

### Renal morphology

Conventional paraffin embedding, 4 μm slices, PAS staining, and preparation of electron microscope. Blue granular were regard as positive areas. Renal damage was viewed by light microscopy. glomerulosclerosis was defined as capillary lumen collapse and/or vitreous changes. Thirty glomeruli were observed in each slice at least. According to semi-quantitative method of Raij et al., glomerulosclerosis index (GSI) was calculated. Graded from 0 to 4 in line with the proportion of sclerosis in glomerulus (0 = 0%, 1 = 1–25%, 2 = 26–50%, 3 = 51–75%, 4 = 76–100%). GSI = [(1*N*l + 2*N*2 + 3*N*3 + 4*N*4)/the total number of glomeruli] × 100%. *N* represents the number of glomeruli in each grade. Blind method was used to reduce error [8].

### Laboratory analysis

To test urinary protein excretion, rats were placed in metabolic cages and urine samples were collected within 24 h. Blood samples were taken from angular vein at the same time. Urinary protein concentration, BUN, and Scr were measured by standard enzymatic method (Boehringer Mannheim, Milan, Italy).

### Immunohistochemical analysis of Col-IV, TGF-β_1_, and TRPC6

After 4 µm sections were collected, the paraffin on the sections was removed by xylene, and then hydrated in graded ethanol. The sections were incubated for 10 min in 10 mmol/L sodium citrate buffer in microwave oven for the purpose of retrieve antigenicity. After that, the sections were incubated with antibody against Col-IV (ab6586 Abcam, Cambridge, MA), TGF-β_1_ (ab92486 Abcam, Cambridge, MA), and TRPC6 (ACC-017, Alomone Labs, Belmont, CA) overnight at 4 °C. The sections were washed fully by phosphate-buffered saline (PBS) solution and incubated with second antibody (Shanghai Changdao, Co., Inc., Shanghai, China) for 30 min. PBS solution was used to replace specific antibody for the purpose of obtaining negative controls. Brownish yellow granular in the glomerulus were considered as positive areas. Semi-quantitative evaluation was performed by Image-Pro Plus6.0 (NIH Image J system, Bethesda, MD).

### Real-time reverse transcription polymerase chain reaction to detect TRPC6 mRNA expression in glomerulus tissue

The total RNA was extracted from the glomerulus tissue according to the Trizol RNA Extraction Kit (Invitrogen, Carlsbad, CA). Primers of TRPC6 and β-actin were designed based on Primer Premier 5.0 (Premier Biosoft, Palo Alto, CA). The primer: F 5′-AGCATCATCGATGCAAATGACAC-3′ and R 5′-CGGTAGGGTCCCACTTTATCCTG-3′ for TRPC6; F 5′-GCCCCTGAGGAGCACCCTGT-3′ and R 5′-ACGCTCGGTCAGGATCTTCA-3′ for β-actin. Each sample was performed in triplicate. The average threshold cycle (the cycles of template amplification to the threshold, CT) was taken as the value of each sample. The data of fold change were analyzed using 2^−△△CT^ method as follows, △CT = CT(TRPC6)-CT(β-actin), △△CT=△CT(TRPC6)-△CT(β-actin) [9].

### Western blot analysis of TRPC6 protein expressions in glomerulus tissue

Total protein was extracted from glomerulus tissue conventionally, and then processed in SDS-PAGE gels. After transferred protein from gels to PVDF membranes, 5% milk blocking buffer was used to block membranes for 1.5 h. After that, membranes was incubation with primary antibodies for TRPC6 (Alomone Labs, Belmont, CA) at 4 °C overnight. β-Actin was used as an internal control. Finally, membranes were incubation with secondary antibody (Abcam, Cambridge, MA) for 1 h at room temperature. Membranes were imaged using LiCor Odyssey scanner (Li-Cor, Lincoln, NE). Protein expression was calculated by ratio (TRPC6/β-actin).

### Statistical analysis

Statistical analysis was performed using SPSS version 16.0 (SPSS, Chicago, IL). Measurement data is expressed as mean ± standard deviation 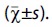 Comparison among groups using single factor analysis of variance or rank sum test, two group comparison using the least significant difference or SNK method. Pearson’s correlation was used to determine the correlations between 24-h urinary proteins and TRPC6 expression. A value of *p* < .05 was considered as a significant difference.

## Results

### Renal morphology and laboratory results

**Figure 1.**
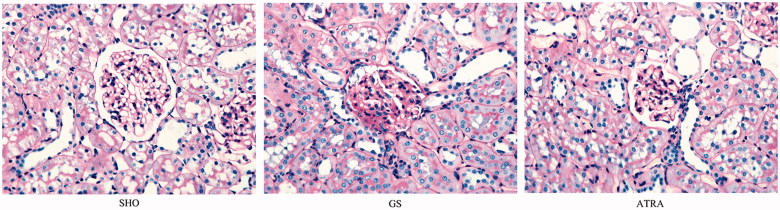
Morphological characteristics of glomerulus under light microscopy in three groups. Collagen deposition was markedly observed in the GS group when compared with the SHO group. The pathological changes in the ATRA group were remarkably reduced when compared with those in GS group. SHO: sham operation group; GS: GS model group; ATRA: GS model group treated with ATRA. All PAS, ×400.

PAS staining showed that the glomerular structure of the SHO group was normal, the capillaries had no congestion, and no tubular type was found in the renal tubular. GS group present as glomerular hypertrophy, wall thickening, balloon adhesions, focal segmental glomerulosclerosis; renal tubular expansion, or atrophy, showing a large number of protein tube type; renal interstitial multifocal mononuclear cells. ATRA group present as fewer lesions than the GS group (Figure 1).

**Figure 2.**
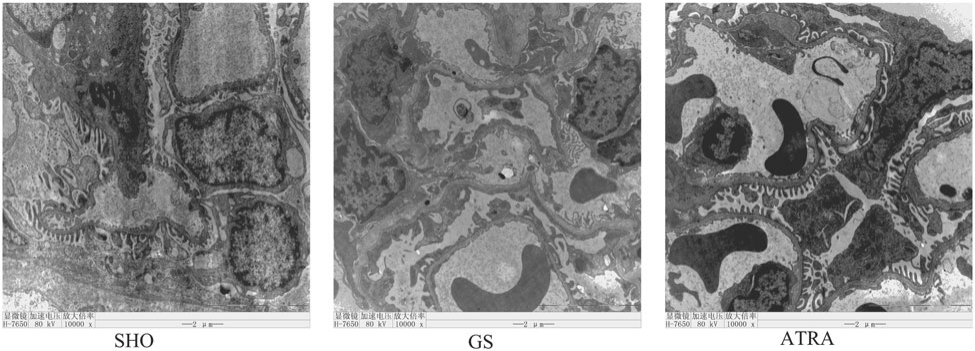
Electron microscopic evaluation of changes in glomerular. The GS group showed that basement membrane thickness varies, foot process diffuse fusion, disappear. The ATRA group presents fewer lesions than the GS group. Magnification 30000×.

Electron microscopic observation of SHO group showed basement membrane normally, foot process completely, endothelial cell pore distribution uniformly. The GS group showed that thickness of basement membrane varies, foot process diffuse fusion, disappear. The ATRA group presents as fewer lesions than the GS group (Figure 2).

The score of GSI in the GS group was significantly higher than that in the SHO group (*p* < .05), and the GSI in the ATRA group was significantly lower than that in GS group (*p <* .05).

At the beginning of the experiment, the 24-h protein excretion was quantitatively located at the same baseline, no statistically significant difference among groups. At the end of 12 weeks, proteinuria in the GS and the ATRA group was significantly higher than those in the SHO group (*p* < .05). The ATRA group was lower than that of the GS group (*p* < .05). There was no significant change of proteinuria in the SHO group during the whole experiment (Table 1).

**Table 1.** Biochemical parameters in each group.

		Scr (μmol/L)		BUN (mmol/L)		24-h proteinuria (mg)	
Group	*N*	0 week	12 weeks	0 week	12 weeks	0 week	12 weeks
SHO	20	34.7 ± 2.3	35.2 ± 3.2	7.1 ± 0.7	7.6 ± 0.8	9.5 ± 2.8	10.5 ± 3.1
GS	20	35.1 ± 2.3	56.6 ± 4.2*	7.4 ± 0.5	13.9 ± 1.5*	8.7 ± 2.3	51.7 ± 7.2*
ATRA	20	35.4 ± 2.7	43.7 ± 4.1*△	7.6 ± 0.6	7.9 ± 1.3*△	8.8 ± 2.1	26.8 ± 3.9*△

* *p* < .05, significantly different from the SHO group at the same time.

△ *p* < .05 significantly different from the GS group at the same time.

There were no significant differences in Scr and BUN among groups at the beginning. At the end of 12 weeks, Scr and BUN in the GS group and the ATRA group were significantly higher than those in the SHO group (*p* < .05). Scr and BUN in the ATRA group were significantly lower than those in the GS group (*p* < .05) (Table 1).

### *Glomerular Col-IV, TGF-β_1_*, *and TRPC6 immunohistochemical staining*

**Figure 3.**
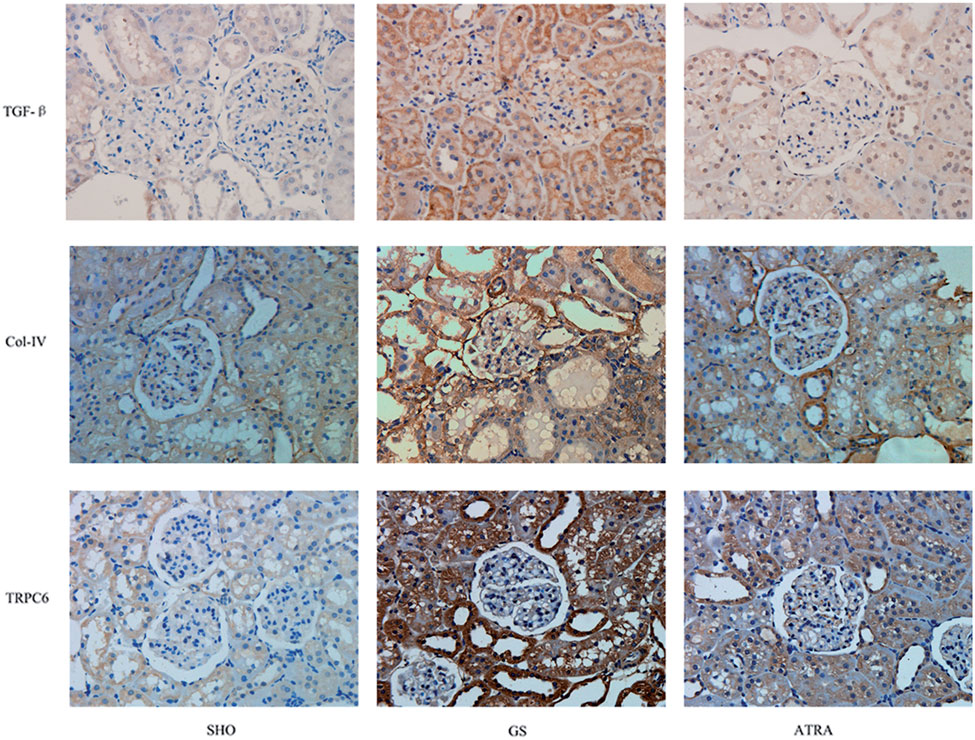
The typical immunohistochemical staining of TGF-β, Col-IV and TRPC6 in glomerulus in three groups. Stainings for TGF-β, Col-IV, and TRPC6 in the GS group were markedly increased when compared with those in the SHO group. The positive stainings of TGF-β, Col-IV, and TRPC6 in the ATRA group were markedly reduced when compared with the GS group. SHO: sham operation group; GS: GS model group; ATRA: GS model group treated with ATRA. Magnification 400×.

Immunohistochemical staining for Col-IV, TGF-β_1_, and TRPC6 was performed. Compared with the SHO group, the levels of TRPC6, TGF-β_1_, and Col-IV in the GS group were all significantly increased (*p* < .05), and the positive staining of TRPC6, TGF-β_1_, and Col-IV in the ATRA group was significantly lower than that in the GS group (*p* < .05). TRPC6 mainly distributed in the podocyte, mesangial area, glomerular capillary loop, and renal tubular epithelial cell cytoplasm. Renal medulla expresses TRPC6 weakly (Figure 3).

### Glomerular TRPC6 mRNA expression

Our studies showed that TRPC6 mRNA expression significantly increased in the GS group than in the SHO group (*p* < .05). The expression of TRPC6 mRNA in the ATRA group was significantly lower than that in the GS group (*p* < .05).

### Glomerular TRPC6 protein expression

**Figure 4.**
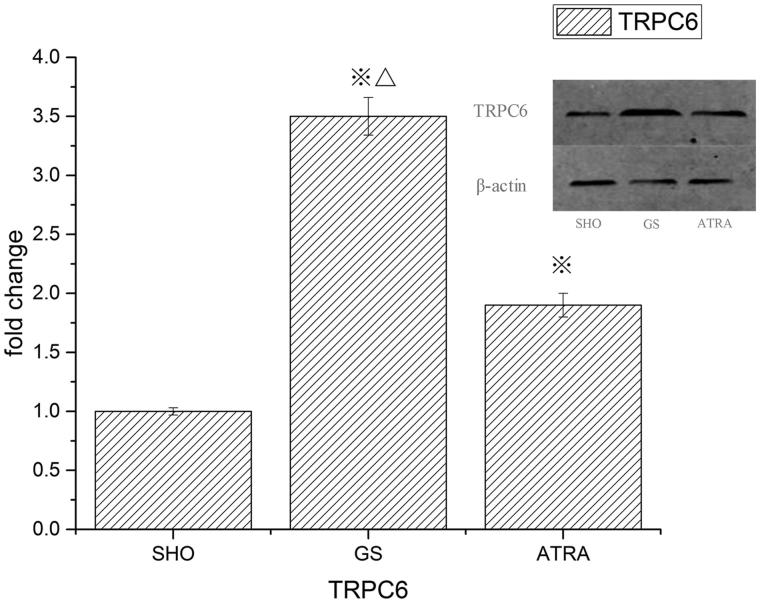
Evaluation of protein expression of TRPC6 (Western-blot). ※ *p* < .05 compared with the SHO group, △ *p* < .05 compared with the ATRA group.

Western blot analysis showed that TRPC6 have a specific band at 106 kDa. The expression of TRPC6 protein in the GS group was significantly higher than that in the SHO group (*p* < .05). The amount of TRPC6 in the ATRA group was significantly lower than that in the GS group (*p* < .05) (Figure 4).

### Correlation analysis

Pearson correlation analysis showed that 24-h urinary protein was positively correlated with TRPC6 protein (*r* = 0.843, *p* < .05).

## Discussion

Glomerulosclerosis is a common pathological stage of many end-stage renal diseases [10]. Proteinuria is a clinical signature of glomerulosclerosis and an independent risk factor for renal disease progression. Multiple mechanisms implicated in the development of proteinuria in glomerulosclerosis, such as oxidative stress and renin–angiotensin–aldosterone system [11,12]. However, the relative factors involved in glomerulosclerosis pathological process have not been fully elucidated. Therefore, it is necessary to provide a promising new insight and effective strategies for the prevention and treatment of glomerulosclerosis.

Adriamycin-induced rat kidney disease model is one of the best models to mimic pathological process of human nephropathy [13]. After a long term of adriamycin injection, pathological changes can occur in a human-like focal segmental glomerulosclerosis. Our results showed that on the 12th week after injection, serious proteinuria, increased BUN, and Scr were evident in adriamycin-induced rats. Light microscopy observations demonstrated glomerular capillary congestion, focal segmental glomerulosclerosis, and dense proteinaceous casts in the ADR rat. Electron microscopy demonstrated focal fusions and widespread effacements of the FPs. In order to further confirm the pathological damage, we examined the expression of glomerular TGF-β_1_ and Col-IV since both of them play an important role in the process of glomerulosclerosis. As a result, immunohistochemistry showed that glomerular TGF-β_1_ and Col-IV was significantly increased in the GS group. Furthermore, calculated glomerular sclerosis index in the GS group was significantly higher than that in the SHO group. Based on these results, we had successfully established the chronic nephropathy model that was similar to human focal segmental glomerulosclerosis induced by adriamycin.

Transient receptor potential channel protein C family includes seven highly conserved Ca^2+^ cation channels. TRPC6 is expressed in a wide range of organs, including kidney [14]. Functional mutations of TRPC6 have been identified as a cause of hereditary FSGS, and elevated expression of TRPC6 was found in several forms of acquired human proteinuric diseases, including minimal change disease, FSGS, and membranous GN [5,15–17]. These findings led to the hypothesis that increased channel expression, possibly via increased TRPC6-mediated calcium signaling, causes glomerular damage. Studies by Krall et al. confirm this hypothesis, that in a transgenic mice, overexpressing TRPC6 in kidney podocytes displayed proteinuria and glomerular injury that similar to human FSGS [18]. Moreover, numerous studies have documented that TRPC6 partially colocalizes with podocyte proteins such as nephrin and podocin [19,20]. TRPC6 might interact with podocin which affect glomerular filtration process by detects mechanical forces, resulting in TRPC6 activation in a cholesterol-dependent manner [21]. Furthermore, in a proteinuria rat model, over expression of TRPC6 was observed accompanied by proteinuria [22]. The present study demonstrated that the overexpression of glomerular TRPC6 in adriamycin nephropathy progression in ADR rats was accompanied by increased proteinuria, a marker of renal injury. Thus, our findings corroborate previous findings. Furthermore, the above changes were correlated with deterioration of BUN and Scr and foot process effacement.

ATRA as a vitamin A derivative has extensive biological effects, such as cell proliferation, differentiation, and apoptosis, and has already widely used in acute promyelocytic leukemia, hyperplastic skin disease treatment [23–25]. In renal tissue, previous studies have demonstrated that ATRA can delay the progression of multiple renal diseases, including membranous nephropathy, minimal change disease, FSGS, and lupus nephritis [26–28]. In this research, we showed that ATRA therapy can delay the progression of glomerulosclerosis in rats. After ATRA treatment, the GSI, pathological changes under light microscope and electron microscope, 24-h proteinuria, and the expression of TGF-β_1_ and Col-IV in glomeruli were significantly improved. This is consistent with our previous study [7]. However, the effect of ATRA on the expression of TRPC6 in glomeruli has not been reported. We first reported that TRPC6 is involved in the effects of ATRA attenuate proteinuria in ADR rats. Our study showed that the expression of glomerular TRPC6 was significantly increased when glomerulosclerosis occurred. After ATRA treatment, the expression of glomerular TRPC6 was significantly descended. Furthermore, glomerular TRPC6 expression levels are closely related to the severity of proteinuria. Our results suggest that the efficacy of ATRA treatment in reducing renal proteinuria is closed related to its regulative effect on TRPC6. ATRA may activate downstream factors by blocking TRPC6 signal, so be able to maintain the structural and functional integrity of glomerular filtration barrier, and play a protective role in antiproteinuria and glomerulosclerosis.

However, it is currently not known how TRPC6 influences glomerulosclerosis, a growing body of evidence points out that harmful intracellular effects caused by increased cation flux cannot be ignored. This belief is not only as a mechanism of renal disease but also prevalent in models of other disease, such as cardiac disease [29]. We assume that activated TRPC6 feels the damage signal in adriamycin-induced glomerulosclerosis, and then activates downstream factors, causing the disappearance of the foot process, then changes in the glomerular filtration barrier, ultimately led to the occurrence of proteinuria. Therefore, pharmacologic TRPC6 blockade is a reasonable therapeutic option. Future research will focus on the mechanisms by which TRPC6 specific affects the pathophysiology of the renal disease.

In summary, our study indicates that TRPC6 involved in the signal cascade of ATRA treating adriamycin induced glomerular injury. ATRA may alleviate proteinuria by down-regulating TRPC6 expression in nephropathy rats. Our findings enrich the theory of ATRA delaying glomerulosclerosis and provide a theoretical basis for promoting clinical prevention and treatment of glomerulosclerosis. However, it must be pointed out that our findings based only on the *in vivo* data, further *in vitro* studies are required.
